# Impact of Genetic Diagnosis on the Outcome of Hematopoietic Stem Cell Transplant in Primary Immunodeficiency Disorders

**DOI:** 10.1007/s10875-022-01403-5

**Published:** 2022-12-10

**Authors:** Federica Forlanini, Alice Chan, Jasmeen Dara, Christopher C. Dvorak, Morton J. Cowan, Jennifer M. Puck, Morna J. Dorsey

**Affiliations:** 1 Division of Pediatric Allergy, Immunology & Bone Marrow Transplantation, UCSF Benioff Children’s Hospital, University of California, San Francisco, USA; 2 Department of Pediatrics, V. Buzzi Hospital, Università Degli Studi Di Milano, Milan, Italy

**Keywords:** Primary immunodeficiency, genotype, transplant, outcome

## Abstract

To evaluate the relationship between knowledge of genetic diagnosis before HSCT and outcome, we reviewed all HSCTs for primary immune deficiencies (PID) performed at UCSF from 2007 through 2018. SCID, a distinct entity identified since 2010 in California by newborn screening and treated early, was considered separately. The underlying genetic condition was known at the time of HSCT in 85% of cases. Graft failure was less frequent in patients with a genetic diagnosis (19% with a genetic diagnosis versus 47% without, *p* = 0.020). Furthermore, event-free survival and overall survival (OS) at 5 years were better for those with a genetic diagnosis (78% with versus 44% without, *p* = 0.006; and 93% versus 60% without, *p* = 0.0002, respectively). OS at 5 years was superior for known-genotype patients with both SCID (*p* = 0.010) and non-SCID PID (*p* = 0.010). There was no difference in OS between HSCT done in 2007–2010 compared to more recently (*p* = 0.19). These data suggest that outcomes of HSCT for PID with known genotype may reflect specific experience and literature, or that a substantial proportion of patients with PID of undetermined genotype may have had underlying conditions for which HSCT may carry greater risk. The higher rate of graft failure in PID with unknown genotype may be in part explained by insufficient conditioning, which in turn could be dictated by compromised organ function in patients undergoing HSCT late in the course. Widespread availability of PID gene sequencing as standard care can provide genetic diagnoses for most patients with PID prior to HSCT, permitting optimization of transplant approach.

**Supplementary Information:**

The online version contains supplementary material available at 10.1007/s10875-022-01403-5.

## Introduction

Primary immunodeficiency (PID) disorders involve a variety of rare genetic defects both intrinsic and extrinsic (e.g., defects in the thymus or stromal cells) to hematopoietic stem cells (HSCs) and their differentiating lineages. More than 400 disorders with 430 different gene defects have been identified by the International Union of Immunological Societies (IUIS) in the last update of human inborn errors of immunity [1]. The recently introduced category of Primary Immune Regulatory Disorders (PIRD) includes disorders with prominent immune-mediated pathology requiring immunosuppression. Management of PID is often complex and may require treatment with hematopoietic stem cell transplant (HSCT) [2].

Over time, advances in genetics, immunology, and public health, including increased availability of genetic sequencing and the introduction of newborn screening (NBS) for severe combined immunodeficiency (SCID), have made possible the identification of new PIDs as well as a deeper knowledge of their molecular pathophysiology [3, 4]. Genetic testing has become an essential part of diagnostic evaluation and management of PID [5]. PIDs caused by genetic defects that result in dysfunction in non-HSC tissue may not benefit from HSCT. Identification of a genetic underpinning is essential to understand utility of HSCT, donor selection, and optimal conditioning regimen [6]. The identification of a genetic defect is indeed critical when considering siblings or parents as potential donors: testing of family members is necessary prior to donor selection to avoid selecting an affected one, or in some situations, a carrier [7]. Early HSCT performed at a young age decreases infectious complications and other comorbidities in most PIDs and therefore remains important for optimal outcomes and long-term survival. [8–11]

We hypothesized an unknown genotype at time of HSCT is associated with a worse outcome. This could be due to a compromised clinical condition of patients who have experienced delays in proceeding to HSCT in the absence of a definitive diagnosis due to hesitation on the part of physicians, parents, and/or 3rd-party payors. Furthermore, the absence of a genetic diagnosis may make it difficult to plan the best approach to HSCT, including conditioning and donor selection [12, 13]. Alternatively, it is possible that well-defined PIDs are diagnosed more readily due to a broader awareness of them, and that available experience and literature contributes to better HSCT outcomes. Therefore, we retrospectively evaluated the relationship between known causative PID genetic mutation before HSCT, and the outcome following HSCT.

## Methods

We conducted a retrospective analysis of all PID HSCTs performed at UCSF Benioff Children’s Hospital for the entire years 2007 through 2018. Data were recorded by individual chart review. This study was approved by the UCSF Institutional Review Board in accordance with the Declaration of Helsinki.

Data analyzed included HSCT characteristics, donor characteristics (matched sibling donors, haploidentical donors, and unrelated donors), genotype, conditioning, Hematopoietic Cell Transplantation-Comorbidity Index (HCT-CI), graft-versus-host disease (GVHD), use of intravenous immunoglobulin infusion (IVIg) before HSCT as well as dependence after HSCT, chimerism, immune reconstitution, all therapy and disease-induced organ toxicities, event-free survival (EFS), and overall survival (OS). In the case of haploidentical-related donors, CD34-selected peripheral blood stem cells (PBSC) were used [14]. Regarding conditioning, all myeloablative regimens consisted of myeloablative targeted doses of busulfan (cAUC of ≥ 60 mg h/L) or ≥ 1200 cGy total body irradiation (TBI); reduced intensity conditioning (RIC) consisted of all other regimens with non-myeloablative targeted busulfan or melphalan. HCT-CI was dichotomized into two categories: “low risk” if HCT-CI = 0 or “intermediate-high risk” if HCT-CI ≥ 1. Second transplants were defined as transplants occurring from a new donor or utilizing a conditioning regimen, and therefore excluded “boost” infusions of HSCs.

PIDs were grouped according to the IUIS phenotypical classification [2, 15]. When patients required > 1 HSCT, the first one was evaluated. SCID NBS in California, implemented in 2010, enabled early diagnosis and treatment of typical and leaky SCID. Therefore, we analyzed this group of patients separately from non-SCID patients. We also divided the population into two time periods, HSCT performed between 2007 and August 2010, and HSCT between September 2010 through 2018, to attempt to address differences in outcomes due to advances in HSCT technique and supportive care, and to consider pre-NBS and post-NBS impact for the SCID patients [16, 17].

We defined a patient as a “known” genotype when the underlying immune defect was diagnosed during pre-HSCT evaluation, and an “unknown” genotype when the genetic defect was not identified before HSCT. We looked for those genes identified as being associated with a PID and pathogenic cause based on the American College of Medical Genetics and Genomics and the Association for Molecular Pathology guidelines [17]. All the genotypes diagnosed in the first time period were reported by the IUIS phenotypical classification of human inborn errors of immunity present at the time [18]. To evaluate if there would have been a difference in outcome if the patient’s diagnosis had been known in the later time period, but not in the earlier one, we looked at patients who received a genetic diagnosis after HSCT. Five patients, for whom the genetic mutation was diagnosed after the transplant, were classified as “unknown” at the time of their first transplant. Genotypes identified after the first transplant were reported by subsequent versions of the IUIS phenotypical classification of human inborn errors of immunity [19, 20]. A patient with Chediak-Higashi syndrome was classified as “known,” even though lacking mutations in the *LYST* disease-causing gene, because the diagnosis was made by the presence of pathognomonic, abnormally large intracytoplasmic granules in neutrophils and consistent clinical findings [21]. Furthermore, we evaluated whether the outcome was different between conditions for which HSCT would be expected to correct the immune dysfunction (e.g., RAG1/2 deficiency) versus immune defects that also involve non-hematopoietic tissues and therefore would not be considered “cured” by HSCT alone (e.g., deficiency of IkBa or adenosine deaminase), and would have a significant effect on survival, morbidity, and a sensitive increase in the complications of high-dose chemotherapy. Lymphocyte subsets and chimerism were followed at 100 days, 6 months, 12 months, and 5 years or at the last encounter post-HSCT. Lymphocyte subsets were assessed by flow cytometry. Normal T cell reconstitution was defined as absolute CD4 + count > 500 × 10^9^/L and CD4/CD45RA count > 200 × 10^9^/L, in addition to the internal UCSF use of > 50% lower limit of reference range response of PBMC or CD45 + cells to phytohemagglutinin. B cell reconstitution criteria included IgM within normal range for age, IgM isohemagglutinin titer ≥ 1:8 dilution, absolute B cell count (CD19 +) > 50/µL, and response to vaccination after discontinuing IVIg. IVIg was restarted if protective antibody titers did not develop following vaccination or if patients experienced recurrent infections.

Demographic, disease-related, and transplant-related variables were evaluated using a *χ*^2^ test or a Fisher exact test for categorical variables, and an ANOVA test or a Wilcoxon Rank Sum Test for continuous variables. Quantitative data were described as median values and interquartile range (IQR), due to their non-Gaussian distribution. The cumulative OS was assessed by the Kaplan-Meyer estimate. For differences between groups, we used the log-rank test. The cumulative EFS probability (considering both deaths and second HSCTs as events) was estimated with life table product limit estimates. Incidence of post-transplant complications, such as GVHD, was estimated by cumulative incidence function for competing risk events. Ninety-five percent confidence intervals (CI) were calculated with the exact Clopper-Pearson test. Statistical significance was set at *p* < 0.05. Statistical analysis was performed with STATA 16 (StataCorp, College Station, TX).

## Results

### Overall Cohort Description and HSCT Characteristics

Ninety-eight patients were included in this study, of whom 66 (67%) were male and 32 (33%) female. Patient characteristics are summarized in Table 1. Transplants were done in the period 2007–2010 for 29 (30%) patients, while 69 (70%) patients were transplanted in the period 2011–2018. The median age at HSCT was 10 months (IQR 3–44). Median follow-up was 6 years (IQR 4–9). Donors included 46 (47%) matched unrelated donors and 52 (53%) related donors (16% matched sibling donors and 36% haploidentical donors). Stem cell sources were bone marrow (BM) in 47 (48%), umbilical cord blood in 6 (6%), and peripheral blood stem cells (PBSC) in 45 (46%) HSCTs. A busulfan-based myeloablative conditioning regimen was used for 41% of HSCTs.

**Table 1:** Patients/HSCT characteristics and genetic identification

Categories		Number of patients (%)	Unknown genotype (%)	Known genotype (%)	*p*-value
*N*		98	15	83	
Age at HSCT, median years (IQR)		10 (3–47)	10 (4–47)	10 (3–44)	0.79
Biological gender	Male	66 (67%)	8 (53%)	58 (70%)	0.21
	Female	32 (33%)	7 (47%)	25 (30%)	
Diagnosis^a^	Bone marrow failure^b^	1 (1%)	0 (0%)	1 (1%)	0.44
	CID	10 (10%)	3 (20%)	7 (8%)	
	CID with syndromic features	16 (16%)	0 (0%)	16 (19%)	
	Congenital defects of phagocyte	3 (3%)	0 (0%)	3 (4%)	
	PIRD	25 (26%)	4 (27%)	21 (25%)	
	SCID	43 (44%)	8 (53%)	35 (43%)	
Donor relation	Related	52 (53%)	8 (53%)	44 (53%)	0.98
	Unrelated	47 (47%)	7 (47%)	40 (48%)	
Source of cells	BM	47 (48%)	7 (47%)	40 (48%)	0.99
	Cord blood	6 (6%)	1 (7%)	5 (6%)	
	PBSC	45 (46%)	7 (47%)	38 (46%)	
ABO incompatibility	No	59 (60%)	7 (47%)	52 (63%)	0.55
	Major	17 (17%)	4 (27%)	13 (15%)	
	Bidirectional	2 (2%)	0 (0%)	2 (2%)	
	Minor	20 (20%)	4 (27%)	16 (19%)	
Conditioning	None	18 (17%)	2 (13%)	16 (19%)	0.95
	Serotherapy only^c^	16 (16%)	2 (13%)	14 (17%)	
	Immunosuppression^d^	2 (2%)	0 (0%)	2 (2%)	
	RIC-busulfan-based (AUC ~ 30)	6 (6%)	1 (7%)	5 (6%)	
	RIC-melphalan-based ^e^	13 (13%)	2 (13%)	11 (13%)	
	MAC-busulfan-based (AUC ≥ 60)	40 (41%)	7 (47%)	33 (40%)	
	MAC-1200TBI-based	3 (3%)	1 (7%)	2 (2%)	
Serotherapy	None	19 (19%)	2 (13%)	17 (21%)	0.52
	ATG	27 (28%)	3 (20%)	24 (29%)	
	Alemtuzumab	52 (53%)	10 (67%)	42 (51%)	
GVHD drug prophylaxis	None	33 (33%)	6 (40%)	27 (33%)	0.60
	MTX + CI	41 (42%)	7 (47%)	34 (41%)	
	MMF + CI	8 (8%)	0 (0%)	8 (10%)	
	Others CI	16 (16%)	2 (13%)	14 (17%)	
CD34 + cell dose (× 10^6/kg), median (IQR)^f^		12.4 (5.6–20)	15.6 (7.2–20)	12.4 (5.4–20)	0.73
Ex vivo T cell depletion		38 (39%)	6 (40%)	32 (39%)	0.92

Overall column with characteristics of all 90 patients

^a^As per the current IUIS phenotypical classification

^b^Dyskeratosis congenita (TINF2)

^c^ATG, *n* = 7; alemtuzumab, *n* = 9

^d^Flu, *n* = 1; Flu/Cy, *n* = 1

^e^with TT, n = 10

^f^For 21 patients, of whom 4 without a genetic diagnosis, we marked the CD34 dose as “not done” as there was a period when for BM products only the total nucleated cell count was performed

Abbreviations: *ATG*, anti-thymocyte globulin; *AUC*, area under curve; *BM*, bone marrow; *CI*, calcineurin inhibitors; *CID*, combined immunodeficiency; *CMV*, cytomegalovirus; *CsA*, cyclosporin; *Cy*, cyclophosphamide; *IQR*, interquartile range; *Flu*, fludarabine; *MAC*, myeloablative conditioning; *MMF*, mycophenolate mofetil; *MTX*, methotrexate; *NMA*, non-myeloablative conditioning; *PBSC*, peripheral blood stem cells; *PIRD*, primary immune regulatory disorder; *RIC*, reduced intensity conditioning; *SCID*, severe combined immunodeficiency; *TBI*, total body irradiation; *TT*, thiotepa

### Genetic Identification

Phenotypes with associated genotypes are listed in Table 2. The most represented diagnoses were in the categories of SCID and PIRD, with 43 (44%) and 25 (26%) patients, respectively. The underlying genetic condition was known at the time of the transplant in 85% of cases. There was a statistically significant difference between the two time periods of HSCT (*p* = 0.005): from 2007 to 2010, we categorized the specific molecular defect prior to HSCT in 20/29 (69%) patients, while from 2011 to 2018 in 63/69 (91%), likely reflecting increased genetic testing availability over time. Of the 15 patients without a genetic diagnosis at the time of the first transplant, as noted in Table 2, we identified the underlying molecular defect after the transplant for 5 of them, with a median time of 14 months (IQR 10–45). There were no differences in age at HSCT between the two eras (*p* = 0.19). There were no correlations in both univariate and multivariate analysis between the identification of the genetic mutation and gender (*p* = 0.20; OR = 0.46, 95% confidence interval (CI), 0.14–1.50, *p* = 0.2), age at HSCT (*p* = 0.79; OR = 0.99, 95% CI, 0.99–1.00, *p* = 0.97), donor relation (*p* = 0.98; unrelated: OR = 1.27, 95% CI, 0.12–13.4, *p* = 0.84), donor category (*p* = 0.92; matched sibling donor: OR 1.82, 95% CI, 0.11–30.8, *p* = 0.67), stem cell source (*p* = 0.99; peripheral blood stem cells: OR = 1.3, 95% CI, 0.15–11.4, *p* = 0.80), HCT-CI score (*p* = ; HCT-CI ≥ 1: OR 1.10, 95% CI, 0.38–71.6, *p* = 0.22), and conditioning regimen (*p* = 0.96; non-myeloablative, OR = 1.16, 95% CI, 0.28–4.85, *p* = 0.83) (Table S1).

**Table 2:** Genotype identified pre-HSCT, and 5 genes identified post-HSCT

Diagnosis	Unknown	Known	Gene
CID	3^a^	7	*ZAP70, CD40L, IL2RG*
CID with syndromic features	0	16	*WASP, RMRP, NFKBIA*
SCID T-B** + **^b^	4^c^	14	*IL7R, IL2RG, JAK3*
SCID T-B-	4^d^	21	*RAG1, RAG2, ADA, DCLRE1C*
Congenital defects in phagocytes	0	3	*ELANE, CYBB*
PIRD	4	21	*FOXP3, SH2D1A, PRF1, UNC13D, RAB27A, IL10RA, PI3KCD, STAT3, XIAP, C1QB*
Dyskeratosis congenita	0	1	*TINF2*
Total	15	83	

^a^Two are now known to be defects in *CD40L* and *MALT1*

^b^It includes both typical SCID and leaky SCID

^c^One is now known to be a non-coding defect in *IL7R*

^*d*^Two are now known to be defects in *RAG1, BCL11B*

Abbreviations: *CID*, combined immunodeficiency; *PIRD*, primary immune regulatory disorder; *SCID*, severe combined immunodeficiency

### Overall Survival and Genetic Identification

**Fig. 1:**
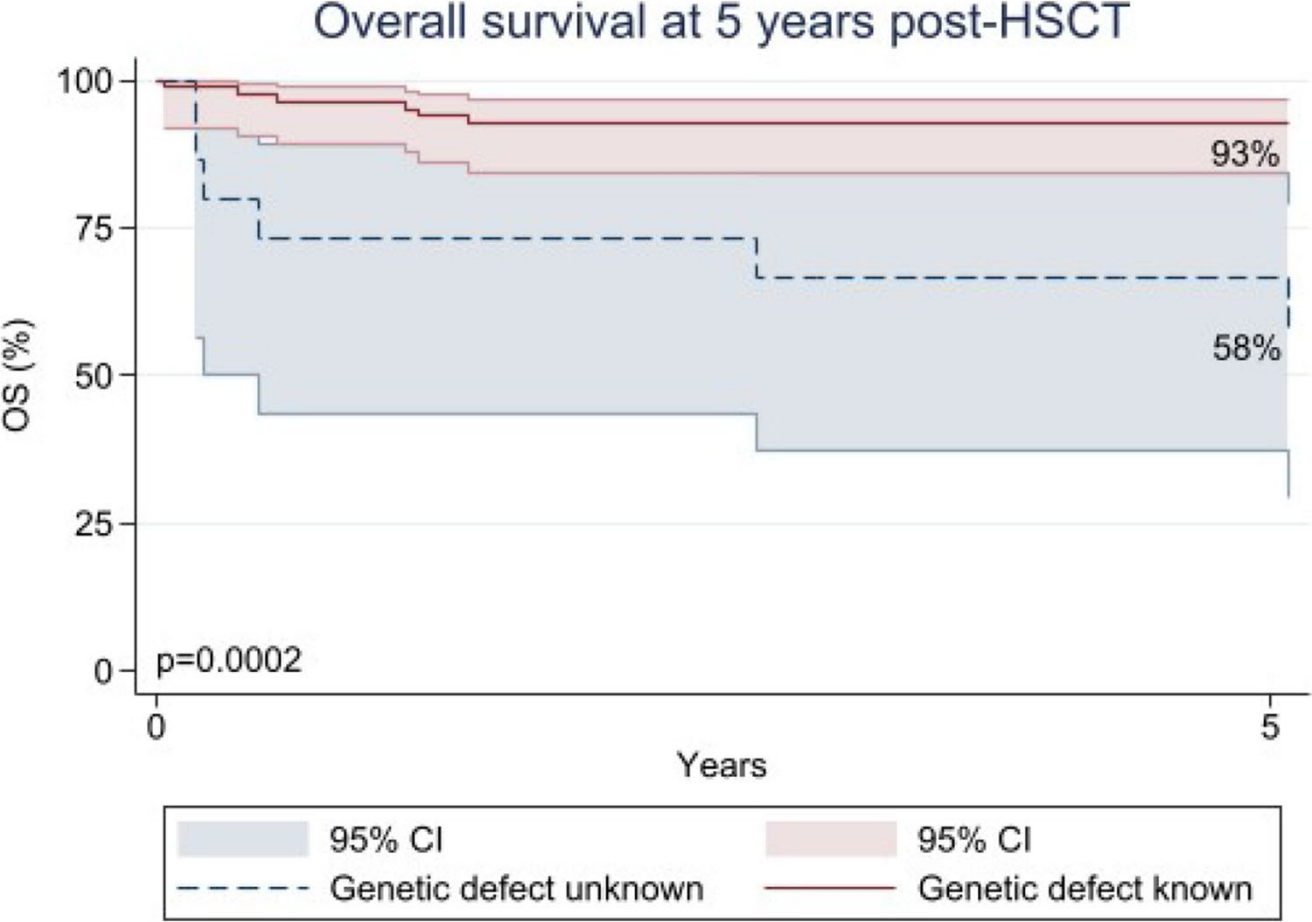
Overall survival at last follow-up by the identification status of the genetic defect

The OS at 5 years post-HSCT was 87% (95% CI, 0.78–0.92). Twelve (12%) patients died, at a median of 185 days after transplant (IQR 75–472). There was a correlation between the identification of the genetic condition before transplant and OS at 5 years or at the last follow-up post-HSCT (*p* = 0.0002), with 58% (95% CI, 0.30–0.79) of patients alive in the group of unknown genetic defect versus 93% (95% CI, 0.84–0.97) in the group with known genetic defect (Fig. 1).

Between the two time periods of HSCT: 79% (95% CI, 0.60–0.90) of patients in the first-period group (2007–2010) vs 91% (95% CI, 0.82–0.96) in the second-period group (2011–2018) survived (*p* = 0.15). A statistical difference in OS by known versus unknown genetic defects was present in both periods. In the first timeframe, 5-year OS was 55% (95% CI, 0.20–0.80) for patients who did not have a known genetic defect versus 90% (95% CI, 0.65–0.97) with a known genetic defect (*p* = 0.032), while in the second time period, 67% (95% CI, 0.20–0.90) of patients who did not have a known genetic defect versus 94% (95% CI, 0.84–0.98) with known genetic defect (*p* = 0.013) were alive, suggesting that knowledge of genetic mutation, rather than advances in HSCT technique or supportive care, is driving the differences in survival.

The mortality rate was 40% (95% CI, 0.16–0.68) in the group without genetic diagnosis versus 7% (95% CI, 0.02–0.15) in the group with genetic diagnosis (*p* < 0.001). In the unknown genotype group, death was attributed to infection in 50% of cases, organ toxicity in 33%, and progressive underlying disease (i.e., HLH reactivation) in the remaining 17%; while in the known group, death was due to infection in 83% of cases and organ toxicity in 17%. There was a significant difference in the 1-year cumulative incidence of organ-toxicity-related death between the two groups, 13% (95% CI, 0.02–0.40) in the unknown versus 1% (95% CI, 0.00–0.06) in the known (*p* = 0.012). Detailed characteristics of deceased patients are provided in supplementary Table S2.

### Event-Free Survival and Genetic Identification

**Fig. 2:**
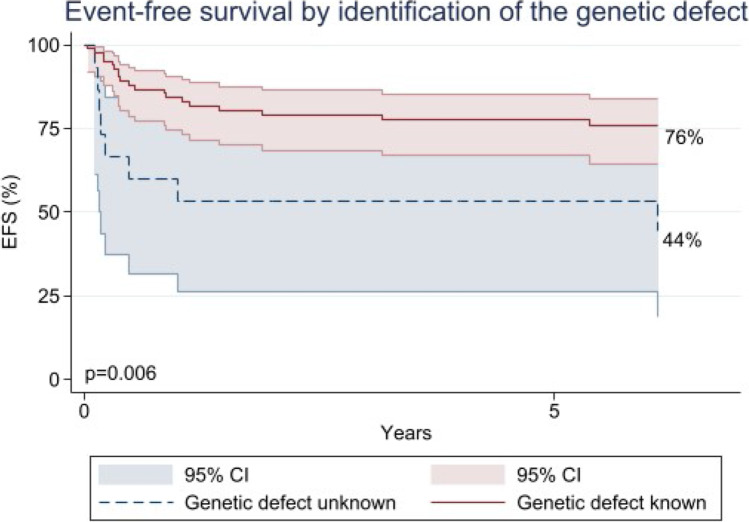
Event-free survival by the identification status of the genetic defect. Events are deaths and second HSCT

Twenty patients (20%) required a second HSCT, mostly due to graft failure (95%), at a median time of 177 days after the first one (IQR 77–583), and of these 15 (75%) were alive at the last follow-up. The EFS at the last follow-up post-HSCT was 74% (95% CI, 0.64–0.82). As shown in Fig. 2, comparing the two groups with and without a genetic diagnosis, EFS at 5 years post-HSCT was influenced by the identification of the underlying molecular defect (*p* = 0.006): EFS for the group without a genetic diagnosis was 44% (95% CI, 0.18–0.68), while for the group with a genetic diagnosis was 76% (95% CI, 0.64–0.84).

There was no difference in EFS at 5 years post-HSCT (*p* = 0.62) by era of transplant: in the first period (2007–2010), the EFS was 66% (95% CI, 0.45–0.80), while in the second period (2011–2018) was 73% (95% CI, 0.60–0.82). In the first period, for the group without a genetic diagnosis, the EFS at 5 years post-HSCT was 44% (95% CI, 0.14–0.72) versus 75% (95% CI, 0.50–0.88) for the group with (*p* = 0.12); in the second period, the EFS at 5 years post-HSCT was 50% (95% CI, 0.11–0.80) for the group without a genetic diagnosis versus 75% (95% CI, 0.61–0.85) for the group with (*p* = 0.03).

### Genetic Identification and Complications Post-HSCT

There was a correlation between the identification of the genetic mutation and graft failure/rejection, seen in 47% (95% CI, 0.21–0.73) of patients without genetic diagnosis versus 19% (95% CI, 0.12–0.29) with (*p* = 0.021). Graft failure was primary in 71% of patients with unknown genetic defects compared with 56% in patients with (*p* = 0.5). Furthermore, we found no significant difference in the proportion with persistent poor graft function requiring IVIg: 6 (86%) patients with unknown genetic defect versus 8 (50%) with (*p* = 0.10).

**Fig. 3:**
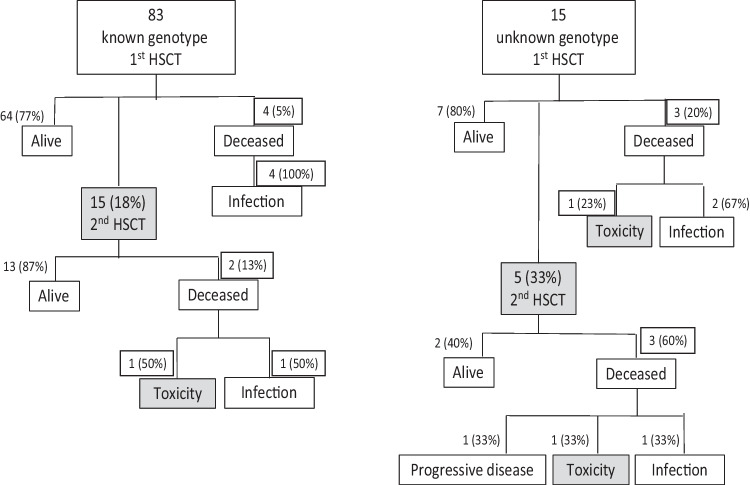
Clinical outcome by the identification status of the genetic defect after 1st and 2nd HSCT

As shown in Fig. 3, five patients (33%) with unknown genetic defects received a second transplant, at a median time of 57 days after the first one (IQR 50–361). Regarding patients with a genetic diagnosis, 15 (18%) received a second transplant, at a median time of 175 days after the first one (IQR 112–489).

After the first HSCT, mortality was 5% (95% CI, 0.01–0.12) for the known genetic group vs 20% (95% CI, 0.4–0.48) for the unknown group (*p* = 0.022); while, after the second HSCT, mortality was 13% (95% CI, 0.16–0.40) for the known genetic group vs 60% (95% CI, 0.15–0.95) for the unknown group (*p* = 0.048). There was no difference between the two time periods for graft failure/rejection (*p* = 0.25).

Other considerations, outside the scope of this study, include the potential for related donors to be carriers of unknown genotype at the time of transplant, thereby leading to unsuccessful HSCT due to potential functional insufficiency of donor cells, although in our cohort we found no significant differences in either OS at 5 years post-HSCT, graft failure or EFS for patients with unknown genotype among those who received a matched sibling donor (possible carriers) versus a matched unrelated donor (not carrier) versus a haploidentical donor (probable carrier for autosomal recessive disorders; possible carrier for female donors to patients with X-linked disorders).

### Genetic Identification and GVHD

As shown in Table 3, of the patients with GVHD, 18 developed acute grades II–IV, at a median of 22 days post-HSCT (IQR 16–63) for a 100-day cumulative incidence of 18% (95% CI 0.11–0.26). Of five patients with chronic GVHD (cGVHD), 2 were extensive in nature, for a 3-year cumulative incidence of 15% (95% CI 0.01–0.46). No differences were observed between the two time periods for either acute GVHD (aGVHD) (*p* = 0.43) or cGVHD (*p* = 0.60). No differences between the two groups, with a genetic diagnosis and without, were observed regarding aGVHD (*p* = 0.30) or cGVHD (*p* = 0.91).

**Table 3:** Genetic identification and outcome and complications post-HSCT

Variables				Overall	Unknown	Known	*p*-value
*N*				98	15	83	
OS at 5 years or last FU				86 (88%)	9 (60%)	77 (93%)	< 0.001
Death, median days (IQR)				185 (75.5–472.5)	127 (69–980)	304.5 (134–429)	0.87
EFS at 5 years %, 95% CI				74% (0.64–0.82)	44% (0.18–0.68)	76% (0.64–0.84)	0.006
Graft failure			Total	23 (23%)	7 (47%)	16 (19%)	0.021
			Primary	14 (61%)*	5 (71%)*	9 (56%)*	0.50
			Secondary	9 (39%)*	2 (29%)*	7(44%)*	
aGVHD	aGVHD CI grades II–IV, 95% CI			18 (18%, 0.11–0.26)	2 (14%, 0.02–0.36)	16 (19%, 0.11–0.28)	0.30
	aGVHD CI grades III–IV, 95% CI			7 (7%, 0.3–0.13)	1 (7%, 0.00–0.29)	6 (7%, 0.03–0.15)	
aGVHD, median days (IQR)				22 (16–63)	22 (19–2351)	22 (14–63)	0.53
cGVHD	CI of any cGVHD			5 (18%, 0.02–0.46)	1 (52%, 0.25–0.74)	4 (5%, 0.02–0.12)	0.91
	CI of severe cGVHD			2 (15%, 0.01–0.46)	1 (52%. 0.25–0.74)	1 (1%. 0.00–0.06)	
cGVHD, median days (IQR)				203 (167–1194)	2650 (2650–2650)	185 (108–698)	0.14
Infection		CMV		27 (28%)	6 (40%)	21 (25%)	0.24
		EBV		6 (6%)	0 (0%)	6 (7%)	0.28
		HHV6		14 (14%)	1 (7%)	13 (16%)	0.40
		ADV		11 (11%)	1 (7%)	10 (12%)	0.54
		HSV1		3 (3%)	0 (0%)	3 (4%)	0.45
		BKV		7 (7%)	1 (7%)	6 (7%)	0.94
		VZV		1 (1%)	0 (0%)	1 (1%)	0.67
		Other Viral		49 (50%)	6 (40%)	43 (52%)	0.40
		Bacterial		55 (56%)	7 (47%)	48 (58%)	0.42
		Fungal		18 (18%)	3 (20%)	15 (18%)	0.86

^*^These percentages are referring to the total number of graft failure (*N* = 23)

Overall column with characteristics of all 99 patients. Abbreviations: *ADV*, adenovirus; *aGVHD*, acute graft-versus-host disease; *BKV*, BK polyomavirus; *CI*, cumulative incidence; *CMV*, cytomegalovirus; *cGVHD*, chronic graft-versus-host disease; *EBV*, Epstein-Barr virus; *FU*, follow-up; *HSCT*, hematopoietic stem cells transplant; *HHV6*, human herpesvirus 6; *HSV1*, herpes simplex 1; *IQR*, interquartile range

### Immune Reconstitution

In our cohort, we analyzed cell reconstitution when all criteria were available and satisfied. We observed a T cell reconstitution in 19/54 (35%) at 6 months, in 48/69 (69%) at 12 months, and in 66/80 (83%) at the last follow-up. The median time for T cell reconstitution was 245 days (IQR 160–538). There were no correlations between the identification of the genetic mutation and the incidence of T cell reconstitution at 6 months post-HSCT (*p* = 0.15), at 12 months post-HSCT (*p* = 0.72), or at the last follow-up (*p* = 0.14).

B cell reconstitution was documented in 55 patients (56%), at a median time of 365 days post-HSCT (IQR 219–730), and it was obtained for 4 (27%) patients with unknown genetic defect versus 51 (61%) with known genetic defect (*p* = 0.012). As shown in Table 3, no significant correlations were detected between the identification of the genetic diagnosis or lack thereof and the development of post-HSCT infections, including viral (*p* = 0.40), bacterial (*p* = 0.42), or fungal (*p* = 0.86).

### SCID Versus Non-SCID

OS at 5 years or at the last follow-up post-HSCT was correlated with identified genetic causes in both SCID and non-SCID disorders (*p* = 0.002), in both time periods. In the SCID group, 5-year survival was 56% (95% CI, 0.15–0.84) of patients without a genetic diagnosis, versus 94% (95% CI, 0.80–0.98) with genetic diagnosis; in the non-SCID group, 57% (95% CI, 0.17–0.84) of patients without a genetic diagnosis versus 91% (95% CI, 0.79–0.97) with a genetic diagnosis were alive after 5 years. We did not find any SCID versus non-SCID influence on the identification of the genetic mutation prior to transplant: incidence of aGVHD (non-SCID *p* = 0.27; SCID *p* = 0.82) versus cGVHD (non-SCID *p* = N.A.; SCID *p* = 0.91). However, there was a significant correlation between the identification of the genetic defect and graft failure for non-SCID patients (*p* = 0.025), with 43% (95% CI, 0.10–0.81) of patients with unknown genetic defect versus 11% (95% CI, 0.03–0.23) of patients with genetic diagnosis; in contrast, no such correlation was found for SCID patients (*p* = 0.29) (supplementary Table S3).

### Predominant Hematopoietic Cell (HC) vs Combined HC and Non-HC Immune Dysfunction

**Fig. 4:**
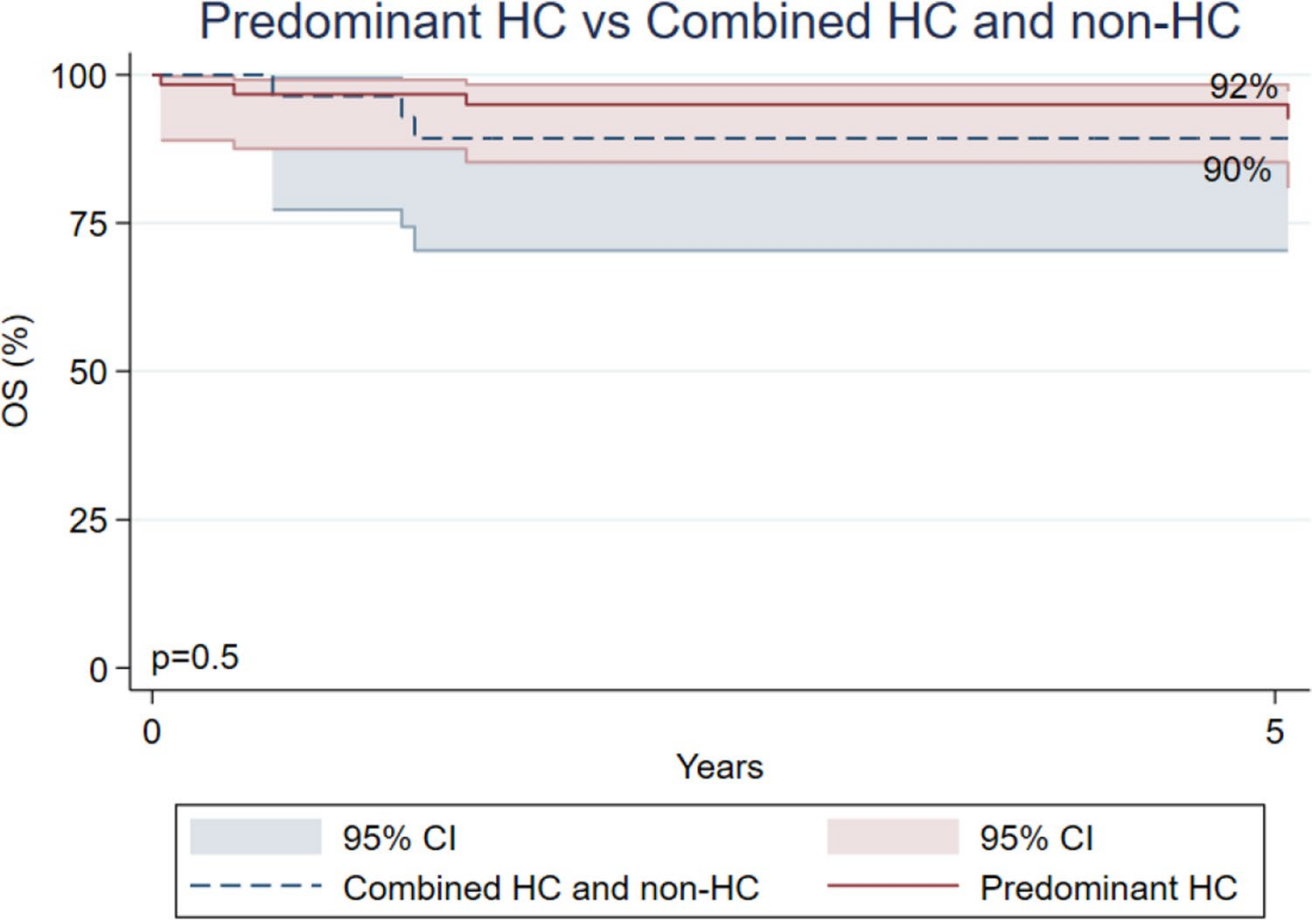
Overall survival at last follow-up in patients with genetic defect in predominant hematopoietic cell (HC) vs combined HC and non-HC immune dysfunction phenotype

In patients with a known genetic defect at the time of the transplant, 57 (69%) had a genetic defect predominantly affecting the HC compartment (supplementary Table S4). As shown in Fig. 4, there was no significant difference in OS at 5 years or at the last follow-up post-HSCT based on the function of the genes affected in this cohort (*p* = 0.5): 25 (90%; 95% CI, 0.70–0.96) patients with genetic diagnoses affecting HC and non-HC immune function versus 57 (92%; 95% CI, 0.81–0.97) patients with genetic diagnoses affecting predominantly HC cells survived. EFS was not influenced by the function of the genes affected in this cohort (*p* = 0.18): EFS was 63% (95% CI, 0.40–0.80) for the combined HC and non-HC group versus 78% (95% CI, 0.63–0.87) for HC predominant group.

## Discussion

This study focuses on the impact of identifying the underlying molecular defect for a primary immunodeficiency disorder prior to HSCT. Considering the genetic heterogeneity of PIDs, finding potential predictors of outcome and success with immune reconstitution is essential [22]. Identification of the molecular defect may facilitate rapid recognition of HSCT candidates, and may inform decisions on donor choice and type of conditioning regimen [9]. Recent SCID studies have highlighted the importance of obtaining a genetic diagnosis and consequently moving towards a classification that allows tailoring of the treatment strategy for each specific genotype [23, 24].

Overall, in this cohort, the underlying genetic condition was known at the time of the transplant in 85% of cases, and we observed an increase in genetic diagnoses over time, with 69% of patients having a genetic diagnosis made between 2007 and 2010, compared to 91% between 2011 and 2018 [6]. This study demonstrated a strong association between known genetic diagnosis and 5-year OS (*p* = 0.0002) in PID recipients of HSCT, and it remained significant regardless of SCID vs. non-SCID diagnosis. These data are consistent with previous studies about PIDs that reported patients with “other T cell deficiencies” having a worse outcome than patients with well-defined SCID [25, 26]. Other authors have emphasized poor survival for patients with undefined PIDs, due to incomplete knowledge of pathophysiology and molecular mechanisms [27, 28]. We did not observe a significant difference between the two time periods of HSCTs despite improvements in the HSCT approach and supportive care. This result may be due to our restricted sample size, as it is in contrast with what Parikh et al. [29] reported in a multicenter cohort study on both primary immunodeficiency and malignancies undergoing HSCT, as well as with another study by Marsh et al. [30] on primary immunodeficiencies.

Furthermore, we observed a higher trend of organ-toxicity-related death in the unknown group: 13% (95% CI, 0.02–0.40) versus 1%. This could potentially be due to underlying DNA-repair defects in some patients with genetically undefined PID. In addition to this, we looked at other possible factors, such as age, gender, donor type, HLA matching, stem cell source, types of infection, and HCT-CI score; however, there were no differences between the groups without and with a genetic diagnosis. Instead, we observed a significant correlation between genotype identification and EFS, with poorer EFS in the undefined genotype: 44% versus 76%. Furthermore, our most interesting finding was the incidence of graft failure in the two groups, unknown (47%) versus known (19%), and a high rate of transplant-related mortality following the second compared to the first HSCT (60% vs 20% in the unknown group; 13% vs 5% in the known). This was responsible for much of the difference in survival between the “unknown” and “known” genotype groups, even if the higher rate of graft failure in PID with unknown genotype was also in part explained by insufficient conditioning. However, we must consider that insufficient conditioning may have been a consequence of the poor health status of the patients since delay in treatment is apt to occur in PID without a genetic diagnosis. Regarding graft failure in the two groups, we found that non-SCID patients were affected the most, with 43% in the unknown group versus 11% in the known group affected. This is potentially because in non-SCID patients the lack of genetic diagnosis makes it difficult to target the best HSCT approach. In some non-SCID PID, the phenotype may be more ambiguous than in typical SCID; therefore, genetic confirmation of the diagnosis is particularly helpful to optimize the management of these patients.

All these findings reinforce our hypothesis that identifying the genetic condition may facilitate the optimization of the transplant approach in terms of donor selection and choice of the conditioning regimen. Known gene diagnosis may help avoid complications from graft failure, thereby resulting in better overall outcomes. Finally, it is important to consider how the identification of a genetic defect might influence the timing of the transplant, as both families and physicians may be apt to pursue HSCT more promptly if a genotype can be identified [7, 31].

This study has some limitations: it is retrospective and includes HSCT with different conditioning regimens. In addition, alternative hypotheses should be considered with respect to the fact that the lack of identification of the genotype has an influence on the post-HSCT outcomes for PID. It is possible that the unknown defects are less treatable with HSCT, since the defects may not be associated with or limited to hematopoietic cells. Thymic defects, including *FOXN1* haploinsufficiency, 22q11.2 deletion, and *TTC7A* deficiency, share many phenotypic features with T lymphopenic HC defects; however, HSCT is not a curative treatment for these patients because HSCs cannot mature without a functional thymus [3, 32–35]. In the setting of most non-HC defects, an HSCT would not be anticipated to benefit the patient, since non-HC immune dysfunctions do not undergo correction with HSCT, and the procedure may induce morbidity and mortality. Nevertheless, five of our patients with unknown genotypes at the time of the transplant eventually had a confirmed genetic diagnosis. This group was too small to manifest a significant difference in either OS or EFS for patients with predominant HC vs combined HC and non-HC immune dysfunction [36]. Another factor to consider is the wealth of experience and literature concerning the treatment of well-characterized PID diagnoses; outcomes may be more likely to be achieved for these, while rarer or still unknown cases of PID will have a higher inherent risk of HSCT. Finally, we need to consider that patients without a genetic diagnosis may have been suboptimally managed for a prolonged period during which infections and/or immune dysregulation have led to chronic organ damage, leading to less successful outcomes. Patients with poor health status may be ineligible for high-dose chemotherapy, which could be the reason for using a suboptimal conditioning regimen and consequent graft failure.

## Conclusion

These data suggest that the lack of an identified underlying genotype impacts post-HSCT outcomes for PID patients which may lead to the need for a second transplant that many patients do not survive. Thus, whole-exome or whole-genome sequencing may be helpful if targeted gene sequencing is unrevealing [37, 38]. Furthermore, novel approaches to testing of intrinsic thymic function are needed [39]. In the era of advanced genetic sequencing, collaborative research and multicenter studies are needed to evaluate PIDs undergoing HSCT and to determine how factors such as donor selection, conditioning regimen, patient health status, and timing of transplant are impacted by the identification of the underlying molecular defect.

## Supplementary Information

Below is the link to the electronic supplementary material.

Supplementary file1 (DOCX 51 KB)

Supplementary file2 (XLSX 178 KB)

## Data Availability

The datasets generated during and/or analyzed during the current study are available from the corresponding author on reasonable request.

## References

[1] Tangye SG, Al-Herz W, Bousfiha A, Chatila T, Cunningham-Rundles C, Etzioni A (2020). Human inborn errors of immunity: 2019 update on the classification from the International Union of Immunological Societies Expert Committee. J Clin Immunol.

[2] Chan AY, Leiding JW, Liu X, Logan BR, Burroughs LM, Allenspach EJ (2020). Hematopoietic cell transplantation in patients with primary immune regulatory disorders (PIRD): a primary immune deficiency treatment consortium (PIDTC) Survey. Front Immunol.

[3] Shamriz O, Chandrakasan S (2019). Update on advances in hematopoietic cell transplantation for primary immunodeficiency disorders. Immunol Allergy Clin North Am.

[4] Amatuni GS, Currier RJ, Church JA, Bishop T, Grimbacher E, Nguyen AA-C, et al. Newborn screening for severe combined immunodeficiency and T-cell lymphopenia in California, 2010–2017. Pediatrics. 2019;143(2):e20182300. 10.1542/peds.2018-2300 PMC6361357 30683812

[5] Chinen J, Lawrence M, Dorsey M, Kobrynski LJ (2019). Practical approach to genetic testing for primary immunodeficiencies. Ann Allergy Asthma Immunol.

[6] Heimall J, Logan BR, Cowan MJ, Notarangelo LD, Griffith LM, Puck JM, et al. Immune reconstitution and survival of 100 SCID patients post-hematopoietic cell transplant: a PIDTC natural history study. Blood. 2017;130(25):2718–27. 10.1182/blood-2017-05-781849 PMC5746165 29021228

[7] Zierhut H, Schneider KW (2014). Stem cell transplantation: genetic counselors as a critical part of the process. Curr Genet Med Rep.

[8] Dvorak CC, Cowan MJ (2008). Hematopoietic stem cell transplantation for primary immunodeficiency disease. Bone Marrow Transplant.

[9] Norman M, David C, Wainstein B, Ziegler JB, Cohn R, Mitchell R (2017). Haematopoietic stem cell transplantation for primary immunodeficiency syndromes: a 5-year single-centre experience. J Paediatr Child Health.

[10] Petrovic A, Dorsey M, Miotke J, Shepherd C, Day N (2009). Hematopoietic stem cell transplantation for pediatric patients with primary immunodeficiency diseases at All Children’s Hospital/University of South Florida. Immunol Res.

[11] Pai S-Y, Cowan MJ (2014). Stem cell transplantation for primary immunodeficiency diseases: the North American experience. Curr Opin Allergy Clin Immunol.

[12] Chinn IK, Chan AY, Chen K, Chou J, Dorsey MJ, Hajjar J (2020). Diagnostic interpretation of genetic studies in patients with primary immunodeficiency diseases: a working group report of the Primary Immunodeficiency Diseases Committee of the American Academy of Allergy, Asthma & Immunology. J Allergy Clin Immunol.

[13] Hagin D, Burroughs L, Torgerson TR (2015). Hematopoietic stem cell transplant for immune deficiency and immune dysregulation disorders. Immunol Allergy Clin North Am.

[14] Dvorak CC, Gilman AL, Horn B, Oon C-Y, Dunn EA, Baxter-Lowe LA (2013). Haploidentical related-donor hematopoietic cell transplantation in children using megadoses of CliniMACs-selected CD34+ cells and a fixed CD3+ dose. Bone Marrow Transplant.

[15] Bousfiha A, Jeddane L, Picard C, Al-Herz W, Ailal F, Chatila T, et al. Human inborn errors of immunity: 2019 update of the IUIS phenotypical classification. J Clin Immunol. 2020;40(1):66–81. 10.1007/s10875-020-00758-x PMC7082388 32048120

[16] Chinen J, Notarangelo LD, Shearer WT. Advances in basic and clinical immunology in 2013. Journal of Allergy and Clinical Immunology. 2014 Apr;133(4):967–76. Bousfiha A, Jeddane L, Al-Herz W, Ailal F, Casanova J-L, Chatila T, et al. The 2015 IUIS phenotypic classification for primary immunodeficiencies. J Clin Immunol. 2015;35(8):727–38.

[17] Richards S, Aziz N, Bale S, Bick D, Das S, Gastier-Foster J (2015). Standards and guidelines for the interpretation of sequence variants: a joint consensus recommendation of the American College of Medical Genetics and Genomics and the Association for Molecular Pathology. Genet Med.

[18] Bousfiha AA, Jeddane L, Ailal F, Al Herz W, Conley ME, Cunningham-Rundles C, et al. A phenotypic approach for IUIS PID classification and diagnosis: guidelines for clinicians at the bedside. J Clin Immunol. 2013;33(6):1078–87. 10.1007/s10875-013-9901-6 PMC4083684 23657403

[19] Bousfiha A, Jeddane L, Al-Herz W, Ailal F, Casanova J-L, Chatila T, et al. The 2015 IUIS phenotypic classification for primary immunodeficiencies. J Clin Immunol. 2015/10/07 ed. 2015;35(8):727–38. 10.1007/s10875-015-0198-5 PMC4659854 26445875

[20] Picard C, Bobby Gaspar H, Al-Herz W, Bousfiha A, Casanova J-L, Chatila T, et al. International Union of Immunological Societies: 2017 Primary Immunodeficiency Diseases Committee report on inborn errors of immunity. J Clin Immunol. 2017/12/11. 2018 Jan;38(1):96–128. 10.1007/s10875-017-0464-9 PMC5742601 29226302

[21] Lozano ML, Rivera J, Sánchez-Guiu I, Vicente V (2014). Towards the targeted management of Chediak-Higashi syndrome. Orphanet J Rare Dis.

[22] Haddad E, Logan BR, Griffith LM, Buckley RH, Parrott RE, Prockop SE, et al. SCID genotype and 6-month posttransplant CD4 count predict survival and immune recovery. Blood. 2018/08/28. 2018 Oct 25;132(17):1737–49. 10.1182/blood-2018-03-840702 PMC6202916 30154114

[23] Haddad E, Hoenig M (2019). Hematopoietic stem cell transplantation for severe combined immunodeficiency (SCID). Front Pediatr.

[24] Dvorak CC, Haddad E, Buckley RH, Cowan MJ, Logan B, Griffith LM, et al. The genetic landscape of severe combined immunodeficiency in the United States and Canada in the current era (2010–2018). J Allergy Clin Immunol. 2018/09/05. 2019 Jan;143(1):405–7. 10.1016/j.jaci.2018.08.027 PMC6322970 30193840

[25] Gennery AR, Slatter MA, Grandin L, Taupin P, Cant AJ, Veys P (2010). Transplantation of hematopoietic stem cells and long-term survival for primary immunodeficiencies in Europe: entering a new century, do we do better?. J Allergy Clin Immunol.

[26] Antoine C, Müller S, Cant A, Cavazzana-Calvo M, Veys P, Vossen J (2003). Long-term survival and transplantation of haemopoietic stem cells for immunodeficiencies: report of the European experience 1968–99. The Lancet.

[27] Yi ES, Choi YB, Lee NH, Lee JW, Sung KW, Koo HH (2018). Allogeneic hematopoietic cell transplantation in patients with primary immunodeficiencies in Korea: eleven-year experience in a single center. J Clin Immunol.

[28] Rousso SZ, Shamriz O, Zilkha A, Braun J, Averbuch D, Or R, et al. Hematopoietic stem cell transplantations for primary immune deficiencies: 3 decades of experience from a tertiary medical center. Journal of Pediatric Hematology/Oncology [Internet]. 2015;37(5). Available from: https://journals.lww.com/jpho-online/Fulltext/2015/07000/Hematopoietic_Stem_Cell_Transplantations_for.21.aspx. 10.1097/MPH.0000000000000352 25985240

[29] Parikh SH, Satwani P, Ahn KW, Sahr NA, Fretham C, Abraham AA, et al. Survival trends in infants undergoing allogeneic hematopoietic cell transplant. JAMA Pediatr. 2019/05/06. 2019 May 1;173(5):e190081–e190081. 10.1001/jamapediatrics.2019.0081 PMC6503511 30882883

[30] Marsh RA, Hebert KM, Keesler D, Boelens JJ, Dvorak CC, Eckrich MJ (2018). Practice pattern changes and improvements in hematopoietic cell transplantation for primary immunodeficiencies. J Allergy Clin Immunol.

[31] Zierhut H, Austin J. How inclusion of genetic counselors on the research team can benefit translational science. Sci Transl Med. 2011;3(74):74cm7–74cm7. 10.1126/scitranslmed.3001898 PMC3753290 21411737

[32] Bosticardo M, Yamazaki Y, Cowan J, Giardino G, Corsino C, Scalia G (2019). Heterozygous FOXN1 variants cause low TRECs and severe T cell lymphopenia, revealing a crucial role of FOXN1 in supporting early thymopoiesis. Am J Hum Genet.

[33] Castagnoli R, Delmonte OM, Calzoni E, Notarangelo LD. Hematopoietic stem cell transplantation in primary immunodeficiency diseases: current status and future perspectives. Front Pediatr [Internet]. 2019 [cited 2020 May 7];7. Available from: 10.3389/fped.2019.00295/full. 10.3389/fped.2019.00295 PMC6694735 31440487

[34] Kammermeier J, Lucchini G, Pai S-Y, Worth A, Rampling D, Amrolia P (2016). Stem cell transplantation for tetratricopeptide repeat domain 7A deficiency: long-term follow-up. Blood.

[35] Marcovecchio GE, Bortolomai I, Ferrua F, Fontana E, Imberti L, Conforti E, et al. Thymic epithelium abnormalities in DiGeorge and Down syndrome patients contribute to dysregulation in T cell development. Front Immunol [Internet]. 2019 [cited 2020 May 7];10. Available from: 10.3389/fimmu.2019.00447/full. 10.3389/fimmu.2019.00447 PMC6436073 30949166

[36] Punwani D, Zhang Y, Yu J, Cowan MJ, Rana S, Kwan A (2016). Multisystem anomalies in severe combined immunodeficiency with mutant BCL11B. N Engl J Med.

[37] Stray-Pedersen A, Sorte HS, Samarakoon P, Gambin T, Chinn IK, Coban Akdemir ZH (2017). Primary immunodeficiency diseases: genomic approaches delineate heterogeneous Mendelian disorders. J Allergy Clin Immunol.

[38] Meienberg J, Bruggmann R, Oexle K, Matyas G (2016). Clinical sequencing: is WGS the better WES?. Hum Genet.

[39] Bifsha P, Leiding JW, Pai S-Y, Colamartino ABL, Hartog N, Church JA (2020). Diagnostic assay to assist clinical decisions for unclassified severe combined immune deficiency. Blood Adv.

