# Supplementary material for: Impact of Genetic Diagnosis on the Outcome of Hematopoietic Stem Cell Transplant in Primary Immunodeficiency Disorders
Source: J Clin Immunol. 2022 Dec 10;43(3):636–46. doi: 10.1007/s10875-022-01403-5 (PMC9958161; doi:10.1007/s10875-022-01403-5)
Supplement: Supplementary file 1 — Supplementary file1 (DOCX 51 KB) [file 10875_2022_1403_MOESM1_ESM.docx]

| **Table S1: Multivariate analysis** | | | |
| --- | --- | --- | --- |
|  | **OR** | **95% CI** | **p** |
| **Age** | | | |
|  | 0.99 | 0.99-1.00 | 0.97 |
| **Gender** | | | |
|  | 0.46 | 0.14-1.5 | 0.20 |
| **Donor relation** | | | |
| Related | 1 |  |  |
| Unrelated | 1.27 | 0.12-13.4 | 0.84 |
| **Stem cell source** | | | |
| BM | 1 |  |  |
| UCB | 0.63 | 0.05-7.82 | 0.72 |
| PBSC | 1.3 | 0.15-11.4 | 0.80 |
| **Conditioning regimen** | | | |
| MAC | 1 |  |  |
| None | 0.87 | 0.12-6.30 | 0.89 |
| NMA | 1.16 | 0.28-4.85 | 0.83 |
| **Donor** | | | |
| MUD | 1 |  |  |
| MSD | 1.82 | 0.11-30.8 | 0.67 |
| Haploidentical | 1 (omitted) |  |  |
| **HCT-CI** | | | |
| Low risk (HCT-CI=0) | 1 |  |  |
| Intermediate-High risk (HCT-CI≥1) | 1.10 | 0.38-71.6 | 0.22 |
| **Table S1: Multivariate analysis**  Abbreviations: OR, odds ratio; BM, bone marrow; UCB, umbilical cord blood; PBSC, peripheral blood stem cells; MAC, myeloablative conditioning; NMA, non-myeloablative; MUD, matched unrelated donor; MSD, matched sibling donor; 95% CI, 95% confidence interval | | | |

| **Table S2: Characteristics of Deceased Patients** | | | | |
| --- | --- | --- | --- | --- |
| **Disease** | **Gene** | **Conditions and Immune dysregulation pre-HSCT** | **Causes of death** | **Time of death after HSCT (days)** |
| **Molecular defect unknown at the time of HSCT** | | | |  |
| **PIRD** | 0 | Cor triatriatum, | Respiratory failure | 60 |
|  |  | CMV infection, Airway edema, Pancytopenia |  |  |
| **Omenn syndrome** | 0 | Rash, FTT | AIHA. Sepsis  (*Pseudomonas aeruginosa, Stenotrophomonas maltophilia*) | 27 |
| **CID** | 0 | ALL, chronic lung disease, CMV pneumonia | Human parainfluenza virus | 35 |
| **SCID T-/B-** | *RAG1* | Chronic rotavirus | cGVHD/PE  (Post 2^nd^ transplant) | 960 |
| **PIRD** | 0 | Mild hepatomegaly, pancytopenia, DM | AIHA, HLH flare  (Post 2^nd^ transplant) | 619 |
| **SCID T-B+** | 0 | CMV viremia | CMV disease  (Post 2nd transplant) | 162 |
| **Molecular defect known at the time of HSCT** | | | |  |
| **PIRD** | *STAT3* | FTT, pseudotumor cerebri, ICH, multiple infections, hepatomegaly, nephrolithiasis, AIHA, ITP, Thyroid dysfunction, IBD pancytopenia, Evans syndrome | Sepsis  (*Escherichia coli ESBL)* | 198 |
| **CID** | *CD40L* | Chronic cryptosporidium | Adenovirus | 134 |
| **CID with syndromic features** | *WASP* | Thrombocytopenia, GI bleeding, eczema | Sepsis  *(Legionella pneumophilia)* | 15 |
| **SCID T-/B-** | *DCLRE1C* | Aphthous ulcers | Respiratory failure | 47 |
|  |  |  | (Post 2^nd^ transplant) |  |
| **CID with syndromic features** | *RMRP* | ARDS, Burkitt ‘lymphoma, chronic Parvovirus infection. Asthma | AIHA/EBV-PTLD | 411 |
| **SCID T-B+** | *IL2RG* | Bronchiectasis, OSAS, multiple infections, Cerebral vasculitis | AIHA/Sepsis  (*VRE, Klebsiella pneumoniae*)  (Post 2^nd^ transplant) | 377 |
| **Table S2: Characteristics of the twelve deceased patients**.  Abbreviations: AIHA, autoimmune hemolytic anemia; ALL, acute lymphoblastic leukemia; ARDS, Acute respiratory distress syndrome; CID, Combined Immunodeficiency; cGVHD, chronic graft-versus-host disease; CMV, cytomegalovirus; DM, diabetes mellitus; FTT, failure to thrive; HSCT, Hematopoietic stem cells transplant; ICH, intracerebral hemorrhage; IBD, Inflammatory bowel disease; ITP, Immune thrombocytopenic purpura; OSAS, obstructive sleep apnea syndrome; PE, pulmonary embolism; PIRD, Primary immune regulatory disorder; PTLD, post-transplant lymphoproliferative disorder; SCID, Severe combined immunodeficiency; VRE, Vancomycin-resistant enterococci. | | | | |

| **Table S3: Genetic identification and outcome post-HSCT in SCID versus Non-SCID** | | | | | | | |
| --- | --- | --- | --- | --- | --- | --- | --- |
|  | | **non-SCID** | | | **SCID** | | |
| **Variables** | | **Unknown** | **Known** | **p-value** | **Unknown** | **Known** | **p-value** |
| **N** | | 7 | 48 |  | 8 | 36 |  |
| **Death,** median days (IQR) | | 82 (65-980) | 166 (74-304) | 1.00 | 172 (69-3189) | 472 (429-516) | 0.56 |
| **OS at 5-years or last FU** | | 4 (57%) | 43 (91%) | 0.012 | 5 (63%) | 34 (94%) | 0.010 |
| **Graft failure** | | 3 (43%) | 5 (11%) | 0.025 | 4 (50%) | 11 (31%) | 0.29 |
| **aGVHD** | aGVHD CI grade II-IV | 0 (0%) | 6 (12%,0.05-0.24) | 0.28 | 2 (28%, 0.04-0.61) | 7 (28%, 0.14-0.43) | 0.82 |
|  | aGVHD CI grade III-IV | 0 (0%) | 2 (4%, 0.01-0.13) |  | 1 (16%, 0.00-0.52) | 4 (12%, 0.04-0.25) |  |
| **aGVHD,** median days (IQR) | |  | 22 (16-32) |  | 22 (19-2351) | 38 (13-105) | 0.52 |
| **cGVHD** | Any cGVHD CI | 0 (0%) | 0 (0%) |  | 1 (50%, 0.15-0.77) | 4 (11%,0.03-0.24) | 0.91 |
|  | Severe cGVHD CI | 0 (0%) | 0 (0%) |  | 1 (50%, 0.15-0.77) | 1 (3%,0.00-0.13) |  |
| **cGVHD,** median days (IQR) | |  |  |  | 2650 (2650-2650) | 185 (107.5-698.5) | 0.16 |
| **Use of systemic steroids** | | 5 (71%) | 22 (47%) | 0.22 | 3 (38%) | 15 (42%) | 0.83 |
| **Table S3: Genetic identification and outcome and complications post-HSCT in SCID versus NON-SCID** | | | | | | | |
| Abbreviations: aGVHD, acute graft-versus-host disease; CI, cumulative incidence; cGVHD, chronic graft-versus-host disease; FU, follow-up; HSCT, Hematopoietic stem cells transplant; IQR, interquartile range; OS, overall survival; SCID, Severe combined immunodeficiency | | | | | | | |

| **Table S4: Classification of Predominant Hematopoietic Cell (HC) vs Combined HC and non-HC Immune Dysfunction Genes** |
| --- |
| **Combined HC and non-HC Immune Dysfunction Genes:** |
| *RAB27A, UNC13D, NFKB1A, STAT3* GOF*, RMRP, XIAP, TINF2, C1qB, ADA, DCLRE1C* |
| **Predominant HC Immune Dysfunction Genes:** |
| *ZAP70, CD40L, IL7R, IL2RG, RAG1, RAG2, FOXP3, SH2D1A, PRF1, CYBB, PI3KCD, MALT1, IL10RA, ELANE, JAK3, WASP* |
